# Intermittent Hypoxia-Hyperoxia and Oxidative Stress in Developing Human Airway Smooth Muscle

**DOI:** 10.3390/antiox10091400

**Published:** 2021-08-31

**Authors:** Colleen M. Bartman, Daniel Wasim Awari, Christina M. Pabelick, Y. S. Prakash

**Affiliations:** 1 Department of Anesthesiology and Perioperative Medicine, Mayo Clinic, Rochester, MN 55905, USA; awari.daniel@mayo.edu (D.W.A.); pabelick.christina@mayo.edu (C.M.P.); 2 Department of Physiology and Biomedical Engineering, Mayo Clinic, Rochester, MN 55905, USA

**Keywords:** intermittent oxygen, hyperoxic-induced lung injury, antioxidants, redox signaling, oxidative injury, antioxidative damage, lung, perinatal, neonatal, lung disease, asthma

## Abstract

Premature infants are frequently and intermittently administered supplemental oxygen during hypoxic episodes, resulting in cycles of intermittent hypoxia and hyperoxia. The relatively hypoxic in utero environment is important for lung development while hyperoxia during the neonatal period is recognized as detrimental towards the development of diseases such as bronchopulmonary dysplasia and bronchial asthma. Understanding early mechanisms that link hypoxic, hyperoxic, and intermittent hypoxic-hyperoxic exposures to altered airway structure and function are key to developing advanced therapeutic approaches in the clinic. Changes in oxygen availability can be detrimental to cellular function and contribute to oxidative damage. Here, we sought to determine the effect of oxygen on mitochondria in human fetal airway smooth muscle cells exposed to either 5% O_2_, 21% O_2_, 40% O_2_, or cycles of 5% and 40% O_2_ (intermittent hypoxia-hyperoxia). Reactive oxygen species production, altered mitochondrial morphology, and changes in mitochondrial respiration were assessed in the context of the antioxidant N-acetylcysteine. Our findings show developing airway smooth muscle is differentially responsive to hypoxic, hyperoxic, or intermittent hypoxic-hyperoxic exposure in terms of mitochondrial structure and function. Cycling O_2_ decreased mitochondrial branching and branch length similar to hypoxia and hyperoxia in the presence of antioxidants. Additionally, hypoxia decreased overall mitochondrial respiration while the addition of antioxidants increased respiration in normoxic and O_2_-cycling conditions. These studies show the necessity of balancing oxidative damage and antioxidant defense systems in the developing airway.

## 1. Introduction

### 1.1. Perinatal Oxygen Transition and the Developing Lung

The immature lung of premature infants (<34 weeks gestation) puts them at risk for inadequate oxygenation and ventilation requiring supplemental oxygen (hyperoxia) with or without ventilatory support [1,2]. The goal of supplemental O_2_ is to maintain adequate oxygenation, but hyperoxia in the immature lung poses some concerns. 

For the premature infant, more is not necessarily better: the in utero environment is relatively hypoxic with a fetal hypoxia pO_2_ of 20–30 mm Hg (roughly 3–5% O_2_), compared to both the postnatal environment (150 mmHg or 21% O_2_), and physiological oxygen concentration (e.g., 100 mmHg or 16% O_2_ in the lung alveolus, but this varies widely throughout the body) [3]. Maintaining fetal hypoxia levels is critical for proper lung development in utero but poses a problem when faced with prematurity. Postnatally, infants are exposed to an environment that would be considered hyperoxic, to begin with, and when this transition occurs prematurely, the lungs are not adequately equipped to manage this significant shift from relatively limited to abundant oxygen availability. However, just because proper lung development occurs in a relatively hypoxic environment does not mean maintaining hypoxia in premature infants is beneficial for promoting postnatal lung development. Postnatal chronic hypoxia detrimentally affects alveolar, airway, and pulmonary vascular development that predisposes infants to chronic diseases of the airway for the remainder of their life [4]. Inadequate oxygenation and subsequent hypoxia following prematurity is the reason supplemental O_2_ is administered in the NICU. While interventions are necessary to maintain oxygenation, the opposite end of the spectrum does not come without a cost: hyperoxia from supplemental O_2_ even at moderate levels (<60% O_2_, which aligns with clinical practice to minimize retinopathy [5,6,7]) administered during prematurity has been associated with subsequent development of bronchial airway hyperreactivity within the first year of life [8] with diminished lung function and increased thickening of the airway, both of which have been linked to the development of childhood asthma [9,10,11,12,13,14].

While the extremes of hypoxia and hyperoxia in the context of prematurity can be problematic in the long term, gauging oxygen demand and adjusting supplemental oxygen is not an accurate and highly dynamic science in practice given an immature respiratory control system in premature infants that leads to the well-recognized periodic episodes of apnea and bradycardia. Clinical intervention typically results in oscillations between hypoxia that requires supplemental oxygen, and subsequent hyperoxia for a brief period thereafter, and a stable level of relative normoxia until the next episode of hypoxia. Accordingly, it becomes important to not only understand the impact of hypoxia or hyperoxia per se in the perinatal, immature lung but also hypoxia-hyperoxia.

### 1.2. Intermittent Hypoxia-Hyperoxia

Intermittent hypoxia-hyperoxia (IHH) is an incidental consequence of supplemental O_2_ administration to premature infants in the NICU. Oscillations in oxygen from hypoxic to hyperoxic ranges in an attempt to maintain oxygenation also have their own detrimental effects on lung development and function, unique from chronic hypoxic or hyperoxic effects. Such effects of IHH involve changes to endothelial cell adhesion, cytokine regulation, coagulation and fibrinolysis, vascular tone, and oxidation-reduction pathways [15]. It is clear that targeting and perfecting oxygen supplementation in premature infants will be important to advance therapeutic strategies, but first, we must understand the underlying mechanisms involved in oxygen effects on the lung. There are multiple factors at play in determining how to approach oxygenation of the premature infant, all of which are up for debate: dosing of O_2_, amount of time supplemental oxygen will be administered, and interventions to promote lung development while preventing lung damage from hypoxic, hyperoxic, or IHH damage. However, the impact of IHH, particularly with moderate hyperoxia has not been well-explored. In a neonatal IHH mouse model, long-term changes in pulmonary mechanics (increased airway resistance, elastance, tissue tamping, and decreased compliance in lung function analyses) have been noted compared to neonates exposed to room air [16].

### 1.3. The Paradox of Oxygen and Oxidative Damage in the Neonatal Lung

ROS and antioxidant machinery are both important for maintaining redox homeostasis. ROS are a natural byproduct of mitochondrial respiration and, at regulated levels, serve important physiological functions by their involvement in a variety of pathways important for cellular homeostasis (e.g., MAPK, ERK, PI3K/Akt, NFκB, eNOS, intracellular calcium, HIF1α) [17,18,19,20,21,22,23,24]. ROS can have downstream transcriptional effects primarily by thiol oxidation of redox-sensitive cysteine residues at the DNA-binding sites of transcription factors (e.g., NFκB, AP-1, HIF1α, and P53), preventing transcriptional activity [25,26,27]. Posttranslationally, ROS can affect protein function by direct oxidative modification of sulfur-containing amino acids, leading to structural and therefore functional changes [25]. Additionally, gap junctions have been shown to be regulated by redox status within the cell and furthermore, ROS have been proposed to act as secondary messengers readily able to transverse gap junctions and initiate signaling cascades in adjacent cells [28]. In the lung, studies have shown that dysregulated glutathione and thioredoxin antioxidant systems are involved in airway diseases by affecting alveolar and vascular signaling from both a transcriptional (e.g., aforementioned NFκB, Nrf2, HIF1α) and posttranslational (e.g., polysulfidration, nitrosylation, glutathionylation) level [27,29,30,31]. Furthermore, the levels of cellular ROS and the length of time ROS or antioxidant systems are elevated are determinants of pathway activation or inhibition in terms of redox homeostasis. More acute oxidative stress is more likely to leverage faster mechanisms such as posttranslational modifications of proteins involved in cellular adaptation while more chronic oxidative stress will drive transcriptional changes impacting cell signaling pathways involved in regaining homeostasis. Additionally, too low ROS levels in the cell, for example by excess ROS scavenging, can lead to reductive stress, which may eliminate protective physiological mechanisms involved in maintaining homeostasis during a period of oxidative stress. ROS therefore, at tightly-regulated low levels, is involved in signaling pathways pertinent to proliferation [32], apoptosis [33], inflammatory cascades [17,18], contraction/relaxation [21], and of course oxidative stress [18,19,24]. In turn, too high ROS levels can lead to oxidative stress. ROS only becomes detrimental to cellular function when accumulated in excess and homeostasis of oxidative/antioxidative pathways are disrupted. This has downstream effects on the aforementioned pathways involved in not only maintaining cellular homeostasis but also associated with the development of multiple airway diseases [34].

### 1.4. Balancing Oxygen, ROS, and Antioxidants in the Human Fetal ASM

While multiple cell types contribute to airway disease at any age, airway smooth muscle (ASM) is a vital cell type for airway structure, tone, and contractility. While there has been much focus on ASM in adult asthma, relatively less is known regarding ASM in the perinatal lung. Previous studies including our own have shown that even moderate hyperoxia (40% O_2_) exposure of human fetal airway smooth muscle (fASM; 18–22 week gestation, a period of rapid bronchial growth) results in increased intracellular Ca^2+^ in response to bronchoconstrictor agonists, as well as increased cell proliferation [35] and ECM deposition [36]. Additionally, we previously demonstrated using a neonatal hyperoxia mouse model mimicking the human premature infant experience, that moderate hyperoxia increases ASM thickness, collagen deposition, and detrimentally alters lung function in response to methacholine challenge [37]. There is currently little to no information on the effects of hypoxia or IHH on developing ASM, but the relevance lies in identifying potential targetable mechanisms for attenuating deleterious effects of O_2_. In this study, we focused on reactive oxygen species as a potential mechanism of interest.

Achieving a balance between ROS and antioxidation is important for maintaining cellular homeostasis. Mechanisms underlying the balance between ROS and antioxidants are pertinent to the lung and in particular, the neonatal lung where antioxidant mechanisms are not well established, and oxygen exposure. Lung damage from ROS can occur from both hyperoxic and hypoxic-induced lung injury. This may seem paradoxical since the production of ROS has been shown to be proportional to the amount of oxygen available for mitochondrial consumption [38]. Without or with too little oxygen, i.e., in hypoxia, ROS is generated through superoxide burst at the mitochondria [39,40]. Superoxide burst occurs relatively quickly upon hypoxic exposure and is not a mechanism of ROS production in long-term hypoxic exposures (i.e., mitochondria superoxide burst generates ROS in acute hypoxia and has not been shown to continue or increase during lengthier hypoxic exposures). This O_2_-dependent and time-dependent superoxide burst at the mitochondrial may be an explanation for the production of ROS in both hypoxic and hyperoxia conditions. Furthermore, the immediate superoxide burst upon hypoxic exposure is thought to initiate signaling cascades that involve stabilizing HIF and driving oxidative posttranslational modifications [39,40]. Excess ROS generation is dependent on the level of hypoxia since severe hypoxia or anoxia does not result in increased ROS production, as seen in moderate hypoxia [41,42,43]. In the respiratory system, excess ROS has been shown to increase lipid peroxidation and damage proteins, increase airway inflammation through the release of proinflammatory cytokines, increase signaling pathways involved in inflammatory responses, proliferation, ECM, and Ca^2+^, affect mucus production, and directly affect ASM function [34,44]. Dysfunction of these pathways is all relevant to airway pathophysiology. Furthermore, asthmatic inflammation has been shown to affect mitochondrial respiration and disrupt the balance of ROS and antioxidant machinery, which only potentiates the problem. For example, airway hyperreactivity and proliferation are linked to increased mitochondrial respiration because higher levels of proliferation and cellular activity demand more mitochondrial biogenesis and the production of ATP to keep up with the energy demand of the cell. Additionally, altered mitochondrial fission (mitochondrial fragmentation) and fusion (mitochondrial networks) are linked to airway diseases with increased ROS decreasing mitochondrial fusion, thereby having fewer mitochondrial networks in the cell [34].

Many investigators have used animal models to better understand the effects of O_2_ on the developing airway (Table 1). These studies have identified multiple pathways involved in airway development that are altered by hypoxia, hyperoxia, or IHH exposures. Examples of pathways detrimentally affected by various O_2_ conditions include intracellular calcium regulation, proliferation, mitochondrial fragmentation, mitochondrial respiration, oxidative damage, and inflammatory response, all of which are concomitantly involved in the pathogenesis of airway diseases. Combined, these studies illustrate the paradigm of O_2_ exposure during the neonatal period and provide a glimpse into why more O_2_ is not necessarily better. It appears that this is not a straightforward answer and is most likely context-dependent. It is plausible that insults from hypoxia, ‘relative’ hyperoxia, and IHH are characteristically unique from one another in terms of how each O_2_ condition damages the developing airway. While multiple groups have investigated the effect of hypoxia, hyperoxia, or oscillations of hypoxia and hyperoxia on the whole lung per se (Table 1), the effects in ASM are not known.

In the present study, we exposed human fASM to various levels of O_2_ (5%, 21%, 40%, and IHH) to determine the effect on ROS production, mitochondrial morphology, and mitochondrial respiration. Moderate (40%) O_2_ was used for hyperoxia exposures as we have previously shown this to produce bronchial airway changes, including increased fASM intracellular calcium [35], proliferation [35], and remodeling [36], along with increased lung reactivity and decreased compliance in a neonatal mouse model [45]. We also included the antioxidant N-acetylcysteine (NAC) to determine the effect on mitochondrial morphology and respiration in the context of O_2_. We postulated that O_2_ increases ROS and affects mitochondrial morphology (e.g., mitochondrial branching and mitochondrial branch length), which signifies an inability to adapt to a change in O_2_ levels (i.e., shorter mitochondria with fewer branches are indicative are mitochondrial fission), that subsequently disrupts mitochondrial respiration (i.e., fragmented mitochondria generally favor glycolytic metabolism). The dynamic nature of mitochondrial morphology is a classic representation of the structure-function relationship, so understanding both mitochondrial morphology and function can help link these two mechanistically. Our findings serve as a platform for further investigation into the importance of balancing redox homeostasis in the developing airway, supporting the notion that ‘more is not always better’ particularly regarding O_2_ exposure. We show that hypoxic, hyperoxia, normoxia, and IHH exposures have differential effects on mitochondrial morphology and respiration in human fASM. Furthermore, we show that the addition of antioxidant NAC may disrupt cellular redox homeostasis that may not be beneficial in all O_2_ contexts. These studies provide an avenue for subsequent investigations to understand mechanisms involved in maintaining optimal ROS/oxidative pathways such that therapeutic targets can be established to prevent long-term damage to the neonatal O_2_-exposed airway.

**Table 1:** Effects of O_2_ on Neonatal Lung Development.

O_2_	Model	Effects on Lung	Ref.
Hypoxia	Neonatal rats	Decreased alveolarization Differential regulation of genes involved in the pathogenesis of postnatal lung remodeling	[46]
	Fetal lung explants from rats	Increased branching Increased epithelial proliferation, differentiation Decreased matrix metalloproteinases	[3]
Hyperoxia	Prenatal LPS and postnatal hyperoxia in mice	Decreased alveolarization; Diffuse fibrosis Altered lung function (decreased compliance, increased resistance)	[47]
	Neonatal rats	Increased lung vascular and airway smooth muscle contraction (hyperresponsiveness), potentially through ROS and endothelin pathways	[48]
	Human fASM exposed to increasing concentrations of O_2_	Increased [Ca^2+^]_i_ response to vasoconstrictor agonists; Increased proliferation, decreased apoptosis; Increased mitochondrial fragmentation	[35]
	Mouse lung epithelial cells	Increased senescence Increased glycolysis Increased proliferation Increased DNA damage	[49]
	Mouse lung epithelial cells and neonatal mice	Decreased mitochondrial respiration Increased mitochondrial networks Alveolar simplification in neonates	[50]
	Neonatal rats exposed to hyperoxia +/− vitamin A	Inhaled vitamin A during hyperoxia exposure increased markers of alveolar maturation, decreased lung damage from hyperoxia, and increased surfactant levels	[51]
	Neonatal mice exposed to moderate or severe hyperoxia	Moderate hyperoxia increased ROS formation/oxidative stress more than severe hyperoxia	[52]
	Hyperoxia-injury in neonatal rats +/− caffeine	Increased total glutathione and hydrogen peroxide, oxidative damage, and antioxidant stress response, effects of which were reduced by caffeine administration	[53]
	Neonatal hyperoxia mouse model with or without caveolin-1 scaffolding domain peptide	Moderate hyperoxia increased airway reactivity and decreased compliance Hyperoxia increased airway thickness and ASM mass Caveolin-1 scaffolding domain peptide mitigated effects of hyperoxia on airway	[45]
	Pulmonary epithelial II cells exposed to hyperoxia +/− NAC	Decreased cell viability, increased cell death Increased ROS, NO, inflammatory cytokines Free radical scavengers reversed these effects	[54]
	Human fASM exposed to hyperoxia +/− senolytics	Increased senescence-associated markers, cell cycle checkpoint markers, and DNA damage markers, leading to secretion of inflammatory markers	[55]
	Neonatal rats exposed to hyperoxia +/− caffeine	Hyperoxia decreased lung development, which was improved by the administration of caffeine	[56]
	Neonatal rats	Increased oxidative damage in the lung through increased malondialdehyde and myeloperoxidase Decreased antioxidant superoxide dismutase Increased infiltration of inflammatory cells and proinflammatory cytokines, effects which were partially reversed by placental growth factor inhibition (which was shown to inhibit NFκB signaling)	[57]
	Neonatal mouse model of hyperoxia exposure +/− superoxide dismutase	Hyperoxia increased antioxidant genes via Nrf2 Hyperoxia decreased superoxide dismutase Overexpression of superoxide dismutase rescued hyperoxia effects on the lung	[58]
	Neonatal mouse model of hyperoxic lung injury +/− Nrf2	Mice deficient in Nrf2 had more severe hyperoxic effects including mortality, decreased alveolarization, edema, inflammation, and DNA damage, and tissue oxidation	[59]
Intermittent	Neonatal mice	Increased airway resistance and decreased compliance	[16]
	Primary murine lung endothelial cell cultures	Intermittent hypoxia-hyperoxia had less of an effect on increasing pro-inflammatory cytokine compared to constant hyperoxia Intermittent hypoxia-hyperoxia increased peroxynitate	[15]
	Lung mitochondria of rats	Intermittent decreased in vitro lipid peroxidation, increased GSH/GSSG ratio, and decreased GSSG content seen in constant hypoxia	[60]
	Neonatal rats +/− superoxide dismutase mimetic, superoxide anion, and peroxynitrate scavenger	Intermittent altered biomarkers of angiogenesis and alveolarization that may be targetable by treatment with antioxidants to decrease lung inflammation	[61]
	Neonatal rats	Decreased lung function Decreased alveolarization Decreased total antioxidant capacity	[62]
	Neonatal mice	Decreased HIF1α and markers of angiogenesis	[63]
	Neonatal mice	Decreased alveolarization Decreased pulmonary total/oxidized glutathione ratio Increased carbonyl content Intermittent hypoxia-hyperoxia exacerbates oxidative stress in lung development	[64]

## 2. Materials and Methods

### 2.1. Human Airway Smooth Muscle

Human fASM were enzymatically dissociated from tracheobronchial trees of 18–22-week gestation fetuses following demise, as previously described [35,65]. fASM acquisition is considered exempt by Mayo Institutional Review Board, with samples entirely de-identified, and no involvement of investigators in the acquisition of the tissues. Cells are characteristically smooth muscle, as they express key markers and regulatory elements involved in [Ca^2+^]_i_ regulation [35,36,66]. Cells were cultured in growth medium: DMEM/F12 (Gibco #11039021, Waltham, MA, USA) supplemented with 10% FBS (R&D Systems #S11150, Minneapolis, MN, USA) and 1% antibiotic/antimycotic) before passage 7 [67]. Serum-deprived medium was DMEM/F12 supplemented with 0.5% FBS and 1% antibiotic/antimycotic.

### 2.2. Treatments and Exposures

Human fASM were plated in 8-well chambered coverslip plates (Ibidi #80826, Gräfelfing, Germany) at 5k cells/well. Cells were serum-deprived 24 h prior to assaying. fASM were exposed to either 5% O_2_ (hypoxia; 5% CO_2_, N_2_ used to displace O_2_; sealed humidified chamber and stored in a 37 °C incubator), 21% O_2_ (normoxia; 5% CO_2_, 37 °C humidified incubator), 40% O_2_ (hyperoxia; 5% CO_2_, 37 °C humidified incubator with O_2_ port) or intermittent hypoxia-hyperoxia (IHH; cycling performed using Bold Line Stage Top Chamber Incubator from Okolab that maintains humidity, 5% CO_2_, 37 °C, and O_2_ levels; San Bruno, CA, USA) for 24 h. IHH consisted of cycling 3 h 5% O_2_ and 3 h 40% O_2_. As a control for oxidative stress, fASM in normoxia were treated with 100 mM H_2_O_2_ for 2 h prior to visualization or assay. A subset of fASM treated with H_2_O_2_ also received 10 mM N-acetylcysteine (NAC; Sigma-Aldrich #A7250, St. Louis, MO, USA) for 2 h prior to visualization or assay. Treatments and exposures were done in serum deprivation medium (DMEM/F12 supplemented with 0.5% FBS and 1% antibiotic/antimycotic).

### 2.3. CellROX and MitoTracker Staining, Microscopy, and Mitochondrial Morphology

Human fASM were dyed with 5 uM CellROX Deep Red Reagent (Ex/Em 644/655 nm; Invitrogen #C10422, Waltham, MA, USA) and 400 nM MitoTracker Green FM (Ex 490/516 nm; Invitrogen #M7514, Waltham, MA, USA) for 30 min. Negative controls (only MitoTracker Green, no CellROX) were prepared appropriately. Cells were imaged using an inverted fluorescence microscope (Keyence BZ-X800E; Osaka, Japan). Exposure time for CellROX was determined by using negative control wells. Mitochondrial morphology was measured by first using Image J software, a freeware from NIH, [68] to isolate single cells following correction for background fluorescence. ‘MitoMorph’ is a mitochondrial morphology macro freely available for download and use with Image J. MitoTracker-stained cells were selected in Image J and a threshold was set to visualize individual mitochondrial networks. Images were then run through the MitoMorph macro/plugin in Image J, which analyzes the images for the area, perimeter, circularity, and major/minor axes. MitoMorph uses these measurements to determine form factor, a measurement of mitochondrial branching/interconnectivity (form factor = perimeter^2^/4π*area), and aspect ratio, a measurement of mitochondrial branch length/elongation (aspect ratio = ratio of major to minor axes of the ellipse equivalent to the object) [69].

### 2.4. Seahorse Bioanalyzer Mitochondrial Stress Test

Human fASM were plated at a density of 2 × 10^4^ cells/well in a growth medium using Agilent Seahorse XF24 Cell Culture Microplates (Agilent #100777-004, Santa Clara, CA, USA). After 24 h, cells were exposed or treated accordingly. 24 h later, mitochondrial stress tests were performed using a Seahorse XF_e_ Bioanalyzer (Agilent Technologies, Santa Clara, CA, USA). Seahorse assay medium consisted of XF Base Medium (Agilent Technologies #103334-100, Santa Clara, CA, USA) supplemented with 10 mM glucose, 1 mM sodium pyruvate, and 2 mM glutamine at pH 7.4. Seahorse XFe24 FluxPak sensor cartridge was hydrated for 24 h prior to assaying using Seahorse XF Calibrant Solution (Agilent #102340-100, Santa Clara, CA, USA). Stock mitochondrial stress test reagents were prepared using Sigma-Aldrich compounds (oligomycin: Sigma #75351; FCCP: Sigma #c2920; Antimycin A: Sigma #A8674; Rotenone: Sigma #R8875; St. Louis, MO, USA). The final concentration of mitochondrial stress test reagents are as follows: 1 µM oligomycin, 1.25 µM FCCP, 1 µM antimycin A, and 1 µM rotenone. Seahorse Bioanalyzer protocol is as follows: 3 cycles per compound of 1’ mix, 2’ wait, 3’ measure. Normalization was done by in-situ cell counting using 1 ug/mL Hoechst 33342 Solution (Thermo Scientific #62249, Waltham, MA, USA) and Cytation 5 imaging (BioTek/Agilent, Winooski, VT, USA).

### 2.5. Statistics

All experiments used 3–4 fASM patient samples with technical replicates noted in legends. Data were analyzed using paired *t*-test or one-way ANOVA, where appropriate, in GraphPad Prism 9.0 software (GraphPad, San Diego, CA, USA). Outliers were determined by Grubb’s outlier test. Data are represented as mean +/− SEM and *p* < 0.05 used for statistical significance.

## 3. Results

### 3.1. Hyperoxia and IHH Increase Oxidative Stress in fASM

**Figure 1:**
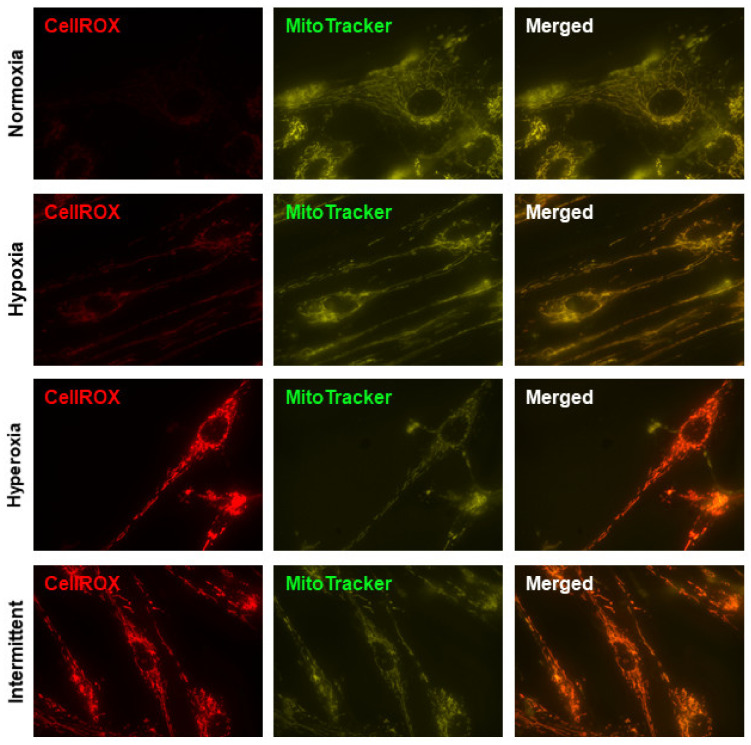
ROS in fASM exposed to normoxia, hypoxia, hyperoxia, or IHH. Human fASM were exposed to either 5% O_2_, 21% O_2_, 40% O_2_, or IHH for 24 h prior to staining with MitoTracker Green FM (mitochondrial marker) and CellROX Deep Red (oxidative stress/ROS marker). Cells were visualized using a Keyence microscopy system. CellROX exposure time was determined using a negative control to establish background exposure. Representative images are shown. Red = CellROX; green = MitoTracker.

CellROX Deep Red, a probe that fluoresces upon oxidation by ROS, was used as a readout of oxidative stress, and MitoTracker Green FM was used to localize mitochondria regardless of mitochondrial membrane potential. Negative control wells were used to determine exposure time for CellROX. Normoxic fASM did not show ROS whereas increased ROS was evident in hypoxic fASM. The most notable increase in ROS was in fASM exposed to either hyperoxia or IHH (Figure 1). These data show fASM are sensitive to various O_2_ exposures by increasing ROS as an indicator of increased oxidative stress.

### 3.2. Mitochondrial Morphology Altered by O_2_ Exposure and NAC

**Figure 2:**
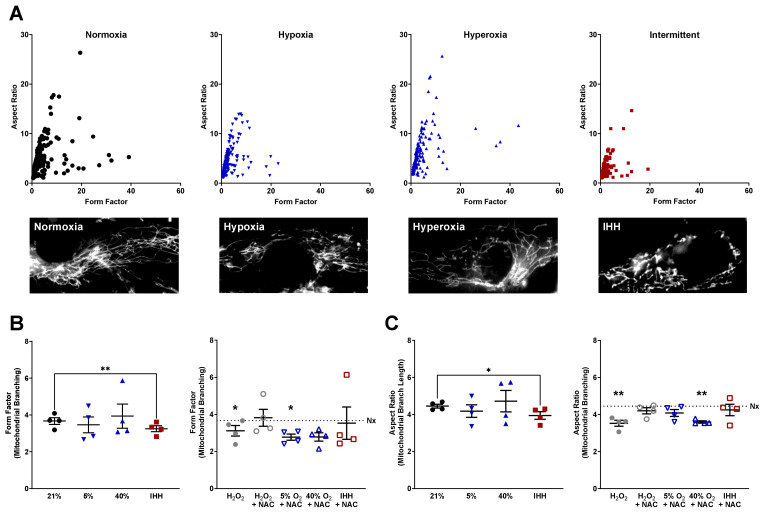
Mitochondrial morphology in fASM exposed to normoxia, hypoxia, hyperoxia, or IHH with or without the antioxidant NAC. Human fASM were exposed to either 5% O_2_, 21% O_2_, 40% O_2_, or IHH with or without NAC for 24 h prior to staining with MitoTracker Green FM. A subset of fASM in normoxia was exposed to H_2_O_2_ or H_2_O_2_ + NAC for two hours prior to staining with MitoTracker Green as a positive control. Cells were visualized using a Keyence microscopy system. ImageJ was used to select individual cells for analysis using a MitoMorph macro and Image J to determine form factor (mitochondrial branching) and aspect ratio (mitochondrial branch length). (**A**) Representative scatter plots of form factor and aspect ratio with representative microscopy images shown of fASM from either 21% O_2_, 5% O_2_, 40% O_2_, or IHH. (**B**) Form factor and (**C**) aspect ratio were measured and analyzed by a MitoMorph macro and Image J. Horizontal dotted lines represent the normoxia control. Data were analyzed using t-tests: * *p* < 0.05; ** *p* < 0.01. Data are represented as mean ± SEM (*n* = 4 fASM patient lines per condition per group).

Accumulation of ROS has been linked to altered mitochondrial morphology [34]. To determine the effect of O_2_ on mitochondrial morphology, fASM were exposed to 5% O_2_, 21% O_2_, 40% O_2_, or IHH for 24 h with or without the ROS scavenger, NAC. A subset of normoxic fASM was treated with H_2_O_2_ with or without NAC 2 h before analysis as a positive control for oxidative damage and antioxidation. Mitochondrial morphology was measured by determining the form factor (mitochondrial branching) and aspect ratio (mitochondrial branch length) of fASM stained with MitoTracker Green FM. Scatter plots combining form factor and aspect ratio analyses enable visualization of mitochondrial morphology with representative images shown (Figure 2A). fASM treated with H_2_O_2_ in normoxia decreased mitochondrial branching (Figure 2B) and branch length (Figure 2C) that was rescued by NAC (Figure 2B,C). In hypoxia, neither mitochondrial branching nor branch length was significantly altered, although NAC decreased branching in hypoxia (Figure 2B). In hyperoxia, mitochondrial branching was not largely altered, but the addition of NAC in hyperoxia decreased mitochondrial branching (Figure 2B) and mitochondrial branch length (Figure 2C). fASM exposed to IHH significantly decreased both mitochondrial branching and mitochondrial branch length compared to fASM in the normoxic condition, an effect rescued by exposure to NAC during IHH cycling (Figure 2B,C). These data show an important role for oxygen exposure and antioxidant defense systems in developing ASM.

### 3.3. Mitochondrial Respiration Altered by O_2_ Exposure and NAC

**Figure 3:**
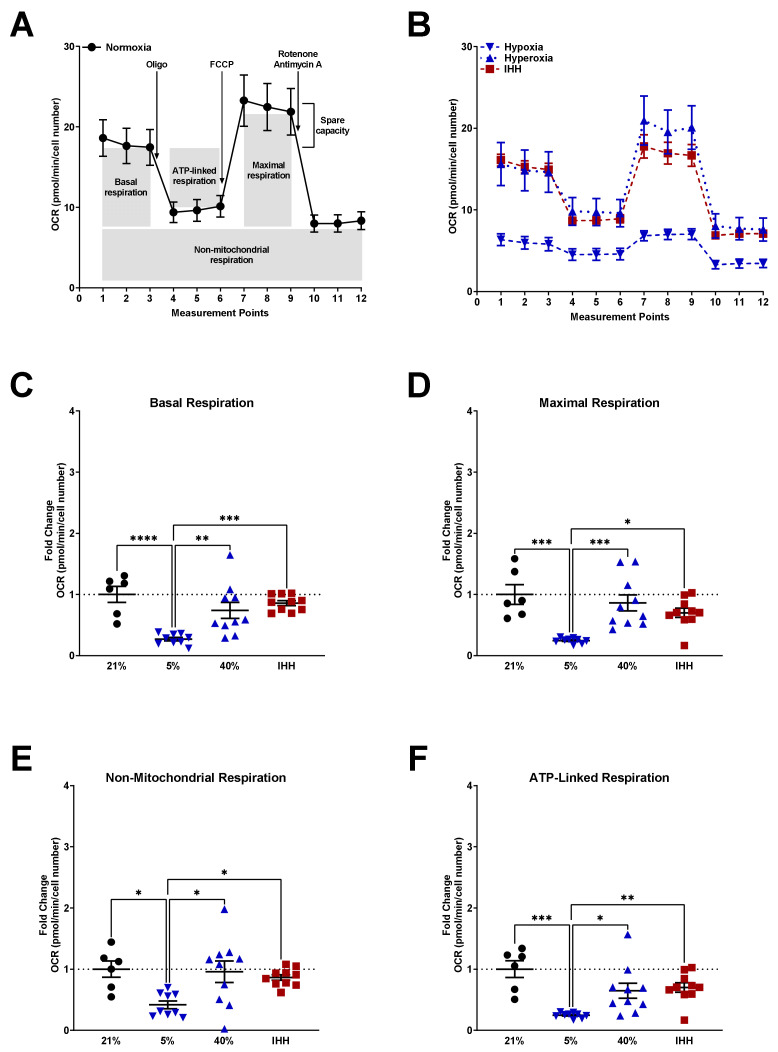
Mitochondrial respiration in fASM exposed to normoxia, hypoxia, hyperoxia, or IHH. Human fASM were exposed to either 5% O2, 21% O2, 40% O2, or IHH for 24 hours prior to mitochondrial stress test using a Seahorse Bioanalyzer. Mitochondrial stress test entails consecutive addition of oligomycin (inhibits ATP synthase), FCCP (uncouples ATP production), and rotenone + antimycin A (complex I and III inhibitors) and is measured by oxygen consumption rate (OCR) over time (measurement points). (**A**) Time-course of mitochondrial stress test from normoxic fASM. (**B**) Time-course mitochondrial stress test from fASM exposed to hypoxia (blue upside-down triangle), hyperoxia (blue right-side-up triangle), and IHH (red square). (**C**) Basal respiration, (**D**) maximal respiration, (**E**) non-mitochondrial respiration, (**F**), and ATP-linked respiration were calculated from Seahorse time measurements. Normoxia = black circles; hypoxia = upside-down blue triangles; hyperoxia = right-side-up blue triangles; IHH = red squares. Data were analyzed using one-way ANOVA: * *p* < 0.05; ** *p* < 0.05; *** *p* < 0.001; **** *p* < 0.0001. Data are represented as mean ± SEM (*n* = 3 fASM patient lines per condition per group; scatter plots include 6–8 technical replicates from three patient lines).

To determine the effect of O_2_ on mitochondrial function, mitochondrial stress tests were performed using a Seahorse Bioanalyzer a, nd measurements of basal, maximal, non-mitochondrial, and ATP-linked respiration was calculated and compared to fASM in 21% O_2_ (Figure 3A; horizontal dotted line in all subsequent graphs). Additionally, fASM were exposed to either 5% O_2_, 40% O_2_, or IHH for 24 h prior to the mitochondrial stress test. Time-course analysis of fASM under various O_2_ conditions showed an overall decrease in oxygen consumption rate (OCR) in hypoxia and IHH (Figure 3B). Basal (Figure 3C), maximal (Figure 3D), non-mitochondrial (Figure 3E), and ATP-linked (Figure 3F) respiration were calculated and represented as fold change from fASM in the normoxia condition. In hypoxia, basal, maximal, non-mitochondrial, and ATP-linked respiration were all significantly decreased compared to normoxia. Hyperoxia had a trending decrease in basal and ATP-linked respiration. IHH had a trending decrease in maximal and ATP-linked respiration (Figure 3).

**Figure 4:**
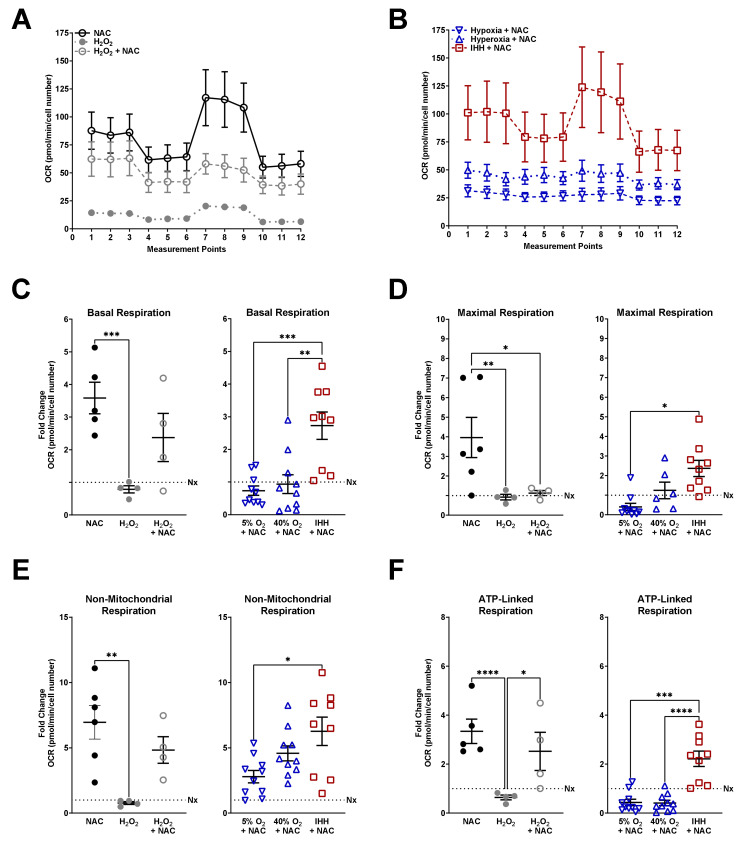
Mitochondrial respiration in fASM exposed to NAC. Mitochondrial stress test entails consecutive addition of oligomycin (inhibits ATP synthase), FCCP (uncouples ATP production), and rotenone + antimycin A (complex I and III inhibitors) and is measured by oxygen consumption rate (OCR) over time (measurement points). (**A**) Time-course of mitochondrial stress tests from normoxia fASM treated with either NAC alone (open black circles), H_2_O_2_ alone (closed gray circles), or NAC + H_2_O_2_ (open gray circles) for 2 h prior to mitochondrial stress test. (**B**) Time-course of mitochondrial stress tests from fASM exposed to either 5% O_2_, 40% O_2_, or IHH treated with NAC for 24 h prior to assaying. (**C**) Basal respiration, (**D**) non-mitochondrial respiration, (**E**) maximal respiration, (**F**), and ATP-linked respiration were calculated from data generated on the Seahorse. NAC = open black circles; H_2_O_2_ = closed gray circles; H_2_O_2_ + NAC = open gray circles; hypoxia + NAC = upside-down open blue triangles; hyperoxia + NAC = right-side-up open blue triangles; IHH + NAC = open red squares. Data were analyzed using one-way ANOVA: * *p* < 0.05; ** *p* < 0.05; *** *p* < 0.001; **** *p* < 0.0001. Data are represented as mean ± SEM (*n* = 3 fASM patient lines per condition per group; scatter plots include 6–8 technical replicates from three patient lines).

A subset of normoxic fASM received three conditions to measure mitochondrial respiration following oxidative damage and/or antioxidation in the context of O_2_: NAC alone, H_2_O_2_ alone, or H_2_O_2_ and NAC (Figure 4). Normoxic fASM receiving NAC increased overall mitochondrial respiration with H_2_O_2_ decreasing overall respiration and NAC providing a moderate rescue from H_2_O_2_ (Figure 4A). fASM receiving NAC in the IHH condition increased overall respiration while NAC in hypoxia or hyperoxia did not respond to mitochondrial stress tests (Figure 4B). Basal (Figure 4C), maximal (Figure 4D), non-mitochondrial **(**Figure 4E), and ATP-linked (Figure 4F) respiration were calculated and represented as fold change from fASM in the normoxia condition (Figure 3A). NAC in normoxic fASM increased basal, maximal, non-mitochondrial, and ATP-linked respiration compared to normoxic fASM without NAC. fASM treated with H_2_O_2_ showed a trending decreased in basal, non-mitochondrial, and ATP-linked respiration. While NAC increased these measurements in the presence of H_2_O_2_, maximal respiration was not increased in H_2_O_2_ + NAC as shown in the NAC alone condition (Figure 4D). In IHH, NAC increased basal, maximal, non-mitochondrial, and ATP-linked respiration.

## 4. Discussion

Understanding mechanisms of O_2_ effects on the developing airway and subsequent risk of life-long chronic airway diseases are important for improving outcomes of former premature infants. In this regard, appreciating that premature infants in the perinatal period are susceptible to episodes of apnea due to an immature central respiratory control system that results in hypoxia, and that clinical interventions involve some level of hyperoxia, overall leading to cycles of intermittent hypoxia and hyperoxia, the mechanisms of IHH in the context of long-term effects on airway become significant. Furthermore, differences between hypoxia, hyperoxia, and IHH become relevant towards understanding whether sustained hyperoxia itself is helpful or not, vs. the reactive implementation of hyperoxia following hypoxia as would occur with IHH. With a focus on bronchial airways and resultant airway hyperreactivity and remodeling, a key cell type becomes the developing ASM. In this study, we show that human fASM are differentially affected by 5% O_2_, 21% O_2_, 40% O_2_, and IHH. Oxidative stress imaging showed increased ROS in hyperoxia and IHH. Mitochondrial morphology analysis showed decreased mitochondrial branching and mitochondrial branch length in IHH while this effect was only evident under hypoxic and hyperoxic conditions when the antioxidant NAC was present. Mitochondrial stress tests showed overall decreased respiration in hypoxia with the addition of NAC increasing respiration in normoxic and IHH conditions. In these studies, there are two ideas that may be counterintuitive at first glance: the notion that ROS can be generated from both hyperoxia and hypoxia and the notion that abundant antioxidant machinery may not always be beneficial in certain contexts (i.e., various O_2_ levels). With this in mind, several questions remain.

Our focus on O_2_ effects on ROS, antioxidants, and mitochondrial morphology and respiration unveils multiple avenues for further mechanistic studies, one of which is the link between altered mitochondrial morphology and respiration. Mitochondrial biogenesis, fission, and fusion are regulated by factors such as mitofusins 1 and 2 (Mfn1, Mfn2), optic atrophy protein 1 (Opa1), fission 1 (Fis1), mitochondrial fission factor MiD49 and MiD51, and dynamin-related protein 1 (Drp1) [70,71]. Due to the dynamic nature of mitochondrial morphology, respiration can be tightly regulated by mitochondrial structure with oxidative metabolism favoring greater mitochondrial fusion and fragmented mitochondria generally favoring glycolytic metabolism [34,72]. Our data show decreased mitochondrial branching and mitochondrial branch length in fASM exposed to IHH, indicating more fragmented mitochondria in oscillating hypoxia-hyperoxia exposures. Interestingly, our data also showed that that the addition of NAC in either hypoxia or hyperoxia decreased mitochondrial branching and mitochondrial branch length that was not evident in either oxygen condition alone. This poses the question of how mitochondrial morphology is regulated in the delicate balance of redox homeostasis. One potential mechanism is that ROS generated from various O_2_ conditions signals downstream to change the expression of mitochondrial biogenesis, fission, or fusion proteins that then regulate mitochondrial morphology. Overloading the system with antioxidants and removing ROS below physiologically important levels may disrupt homeostasis to the point that the cell’s adaptive mechanism involves altering mitochondrial morphology to prevent further damage until homeostasis can be achieved.

Questions remain as to the effects of various O_2_ conditions, including IHH, on mitochondrial calcium regulation. Not only does mitochondrial calcium play a role in mitochondrial respiration, but mitochondrial calcium is also involved in cytoplasmic calcium regulation, which contributes to airway hyperreactivity in diseases such as asthma [73,74]. Additionally, hyperoxia exposure in the developing neonatal airway drives extracellular matrix deposition [36], intracellular calcium concentrations, hyperreactivity, and proliferation [35], and impairs lung function [75,76,77]. It is reasonable to speculate that O_2_ exposure and oxidative damage play an important role in mitochondrial and cytoplasmic calcium regulation. In ASM, calcium release from the sarcoplasmic reticulum drives cellular contraction, and calcium exchange between the mitochondrion and sarcoplasmic reticulum is important for regulating contraction as well as other cellular functions, including ATP production from oxidative phosphorylation and subsequent ROS production [70,78]. Our data showing increased oxidative damage and altered mitochondrial morphology in response to various O_2_ conditions or antioxidants build on our previously published work showing increased intracellular calcium response to agonists in human fASM exposed to hyperoxia [79]. It is now worth investigating whether ROS can be targeted to help mitigate elevated [Ca^2+^]_i_ in various O_2_ conditions.

Another important aspect to consider is the mechanism of ROS production and removal so that specific targets can be identified in designing therapeutic strategies for premature infants administered supplemental O_2_. Superoxides are enzymatically generated from the reduction of molecular oxygen by oxidative phosphorylation, as well as through monooxygenase, NADPH oxidase, and xanthine oxidase. Hydrogen peroxide is subsequently generated from superoxide by superoxide dismutase, an antioxidant enzyme expressed at high levels in the lungs [80]. Other ROS, such as hydroxyl radical, singlet oxygen, and peroxynitrate are produced through non-enzymatic reactions in the cell [81,82,83]. Glutathione peroxidase and glutathione reductase are antioxidant enzymes for hydroperoxides such as hydrogen peroxide [81,84]. In the present studies, we used H_2_O_2_ to induce oxidative stress, which typically occurs through a Fenton reaction in which H_2_O_2_ reacts with Fe^2+^ to produce hydroxide ion and hydroxyl radical. Redox-active metal catalysts such as Fe and Cu are therefore part of ROS generation within the cell and important pathways to consider in future studies on mechanisms of ROS production in various O_2_ conditions [25]. Deciphering the precise role of hypoxic, hyperoxic, or IHH-derived ROS in the premature airway may identify targets for maintaining redox homeostasis in developing ASM.

ROS have also been directly linked to oxygen-sensing pathways through HIF1α [40,85]. Mitochondrial ROS have been shown to inhibit prolylhydroxylase (PHDs), leading to HIF1α stabilization, translocation to the nucleus, and transcription of downstream targets [40]. In pulmonary artery smooth muscle cells, hypoxia was found to increase cytosolic calcium, an effect dependent on mitochondrial ROS production in low O_2_ [40,86]. Whether this is through HIF1α and works through a pathway conserved in airway smooth muscle is unclear. Future studies include determining the effect of ROS from various O_2_ conditions on intracellular calcium regulation in a HIF1α-dependent manner. This pathway would unveil targets for regulating cellular functions in O_2_ conditions.

Sex differences in lung development and the effects of O_2_ on the premature airway should also be considered. There are noted sex differences in lung function from the perinatal period through the neonatal period and adulthood [87]. Males and females exhibit differences in lung development and therefore treatment outcomes. While females tend to be born with smaller lungs on average compared to males, female lungs tend to be more advanced compared to male lung development, and neonatal females are not as high of a risk to develop respiratory distress syndrome as neonatal males [87,88,89]. Studies have shown differences in neonatal hyperoxia exposure based on sex such that neonatal male mice exposed to hyperoxia had increased bronchiolar epithelial thickening while neonatal female mice exposed to hyperoxia had higher bronchiolar collagen [90]. Additionally, neonatal female mice exposed to hyperoxia had lower lung compliance and higher resistance than neonatal male mice exposed to hyperoxia [90]. Understanding sex differences in postnatal lung development and potential differential responses to O_2_, oxidative damage, and stress response will be key to identifying accurate targets for therapies.

## 5. Conclusions

In summary, our studies show that there are multiple facets involved in understanding the effects of O_2_ on the developing airway. Our data highlight these nuances and the need to understand optimal O_2_ dosage and timing to mitigate potential deleterious effects on lung development and changes in mitochondrial structure/function that may predispose infants to lifelong diseases of the airway. For example, while hypoxia did not increase ROS to the same extent as hyperoxia or IHH (nor did hypoxia substantially alter mitochondrial morphology), the addition of the antioxidant NAC under hypoxic conditions decreased mitochondrial branching, suggesting that maintaining appropriate ROS levels is important for cellular homeostasis. Additionally, mitochondrial respiration was overall reduced in hypoxia without an evident benefit of NAC, which may be appropriate considering ROS are important for signaling and adaptation to hypoxia so their removal under hypoxic conditions may cause more harm than benefit. In a similarly nuanced fashion, hyperoxia substantially increased ROS, but mitochondrial morphology was not altered until the addition of NAC. IHH tended to decrease overall mitochondrial respiration with NAC largely rescuing these effects. Importantly, considering IHH incidentally results from supplemental O_2_ aiming to maintain oxygenation in the NICU, it is relevant to compare hypoxia to IHH exposures. Our data suggest IHH may provide protection from the effects of hypoxia, at least in terms of mitochondrial respiration. Furthermore, IHH increased ROS and altered mitochondrial morphology (decreased mitochondrial branching and mitochondrial branch length), the latter of which was rescued by the addition of NAC. These findings support the notion that redox balance is vital for maintaining homeostasis in adaptation to various O_2_ conditions, but this concept is not straightforward for understanding and improving therapeutic strategies. Determining how to use oxygen as a therapeutic approach for premature infants in the NICU has been debated. The dose of oxygen, the frequency of administration, and the length of time of administration, all relative to the stage of lung development in prematurity are important to understand so that strategies can be optimized in the appropriate context. Additionally, the stage of lung development at birth (in particular the extent of prematurity) and existing sex differences may help optimize postnatal lung growth and development to mimic a more natural transition from the prenatal hypoxic environment to the extrauterine relatively hyperoxic exposures. Discovering the mechanisms of ROS production and antioxidant pathways involved so that redox homeostasis can be maintained without “overshooting” in either direction will be key to understanding these targetable pathways. Our studies contribute to the understanding of how O_2_, ROS, and antioxidants affect mitochondrial function and how these factors must be balanced in developing airway smooth muscle. However, future studies will be needed to substantiate the link between O_2_, ROS, antioxidants, and mitochondrial structure/function in the context of the developing airway and susceptibility to disease. Identifying the specific pathways involved in fASM ROS generation and antioxidant defense would enable more mechanistic studies, which could branch to using in vivo models in which neonatal mice are exposed to either hypoxia, hyperoxia, normoxia, or IHH.

## Data Availability

The data presented in this study are available upon request from the corresponding author. The data are not publicly available due to institutional restrictions.
